# Breast Reconstruction Using Laparoscopically Harvested Pedicled Omental Flap: Imaging Findings and a Case of Recurrence Among Eight Patients

**DOI:** 10.2174/0115734056359849241226055644

**Published:** 2025-01-02

**Authors:** Jung Hee Byon, Soyeoun Lim, Kyoungkyg Bae, Minseo Bang

**Affiliations:** 1 Department of Radiology, Ulsan University Hospital, University of Ulsan College of Medicine, 877 Bangeojinsunhwando-ro, Dong-gu, Ulsan 44033, South Korea

**Keywords:** Breast reconstruction, Omental flap, Laparoscopy, Mastectomy, Breast cancer, MRI, Patients

## Abstract

**Background:**

Laparoscopically Harvested Pedicled Omental Flap (LHPOF) has become a viable option for breast reconstruction due to advancements in minimally invasive techniques, offering benefits like reduced postoperative pain and minimal scarring.

**Case Presentation:**

This study examines the imaging findings in eight patients who underwent breast reconstruction using a LHPOF. Imaging modalities, including mammography, ultrasonography, MRI, and CT, consistently showed reconstructed breasts with fat replacing glandular tissue and numerous internal vessels. One case of recurrence was detected, demonstrating the efficacy of conventional surveillance imaging studies in facilitating the detection of recurrences.

**Conclusion:**

This is the first report detailing imaging findings of breast reconstruction using an LHPOF, including a recurrence case. Understanding these imaging results is crucial for effective surveillance in breast cancer patients with omental flap reconstruction.

## 1 INTRODUCTION

Breast cancer is the most common malignancy in women, and it is increasingly affecting younger patients [1]. With advancements in breast reconstruction surgery techniques, the cosmetic expectations of patients have grown significantly.

Among the various reconstructive surgery methods, one approach in autologous tissue transplantation involves using the omental flap. Autologous omental flaps can be transferred either on a pedicle, remaining attached to its own blood supply, or as a free flap, requiring microvascular anastomosis of the donor tissue vessel to the recipient site vessel [2]. The use of the pedicled omental flap for immediate breast reconstruction was first described by Kiricuta in 1963 [3]. Advancements in laparoscopic technology and surgical skills have enabled physicians to harvest the omental flap through minimally invasive procedures [4]. Laparoscopic harvesting of the omental flap involves the dissection and mobilization of the omentum using small incisions, which results in less postoperative pain, shorter hospital stays, and minimal scarring compared to traditional open surgical methods [5]. Some researchers have reported their experience of immediate breast reconstruction using a Laparoscopically Harvested Pedicled Omental Flap (LHPOF) [4-6].

Herein, we report the cases of eight patients who underwent LHPOF for breast reconstruction, including one case of recurrence and detail the multi-modality imaging findings. These results are presented with the aim of improving radiologic understanding during surveillance follow-up.

## 2 CASE REPORT

Eight patients who underwent modified radical mastectomy with a laparoscopically harvested pedicled omental flap between December 2014 and April 2023 were enrolled in this study. The clinical information is listed in Table **1**. Seven patients underwent immediate reconstruction following breast cancer removal surgery, and one patient underwent reconstruction six months later for cosmetic purposes. All patients recovered uneventfully following surgery.

### 2.1 Surgical Procedures

At our hospital, breast reconstruction surgery with an omental flap involved three key steps. The first step involved breast surgery and subcutaneous tunneling for omental transfer. Mammary tissue manipulation, including tumor removal, was conductedcautiously, leaving a 5–7 mm thick flap. Subcutaneous fat was removed for tumors near the skin. Lymph node procedures wereperformed. Subcutaneous tunneling post-surgery minimized bulging.

The second step included omental harvesting *via* laparoscopic surgery and subsequent extraction of the omentum. The greater omentum was dissected along the avascular plane from the splenic to the hepatic flexure. Left gastroepiploic vessels wereligated and resected around the spleen, with their branches dissected along the greater curvature. Right gastroepiploic vessels were identified and preserved as feeding vessels. An epigastric incision below the xiphoid facilitated communication with the tunnel. The pedicled flap was pulled through the subcutaneous tunnel, and the pedicle is intracorporeally fixed to the linea alba to prevent twisting and herniation.

The final step focused on breast reconstruction using the omental flap. Once the omentum was extracted into the mastectomized cavity, the pedicle was securely fixed to the chest wall near the subcutaneous tunnel opening. Subsequently, the harvested omental flap was shaped and carefully placed in the defect space. Special care was taken to secure the epigastric opening and ensure it is neither too narrow nor too wide. Following the meticulous adjustment in the epigastric area, closure of the breast skin incision using absorbable sutures was performed.

### 2.2 Non-recurrence Cases (Cases 1-7)

**Fig. (1):**
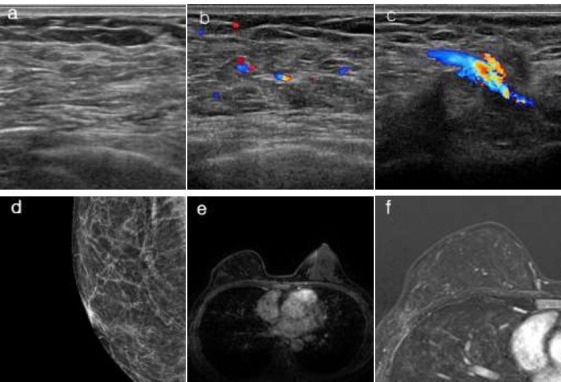
Right reconstructed breast with LHPOF. (**a**, **b**) On ultrasonography, omental fat and numerous internal vessels are observed in the breast. (**c**) On ultrasonography, a pedicled right epigastroepiploic vessel is identified in the subcutaneous tunnel. (**d**) On mammography, omental fat replaces the glandular tissue in the breast. (**e**, **f**) On MRI, the contrast-enhanced and subtraction 2-minute sequence shows the reconstructed breast with omental fat instead of glandular tissue, with an outline that closely matches the contralateral breast.

**Fig. (2):**
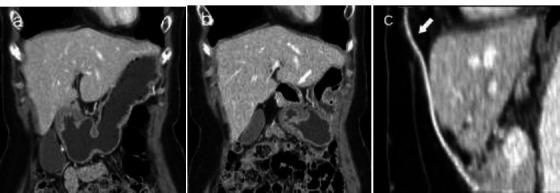
Abdomen CT in a patient who underwent LHPOF. (**a**) Pre-operation. (**b**) Post-operation: The stomach is shifted more towards the midline, and the antrum and duodenal bulb (arrow) is pulled upwards by the pedicled omental flap, compared to the previous CT. (**c**) Post-operation: Curved sagittal MPR (multiplanar reformat) shows the right epigastric vessel (arrow) passing through the tunnel directly beneath the xiphoid process, accompanying the omentum.

For surveillance imaging, mammography, ultrasonography, and MRI were performed in patients who underwent breast cancer surgery. On mammography, compared to the contralateral breast, no glandular tissue was visible, and it was filled with omental fat. On ultrasonography, a fatty lesion with margins that were indistinct from the surrounding fat and internal vessels was observed within the breast. A bulging fatty lesion containing internal vessels was also seen in the midline subcutaneous tunnel. Doppler's study of the vessels within the subcutaneous tunnel revealed that blood flow was without resistance. On MRI, the outer contour of the operated breast was similar to that of the opposite breast. There was no visible glandular tissue, as it had been replaced by omental fat, and numerous blood vessels were observed within the breast. Additionally, there was slight bulging in the subcutaneous tunnel containing an omental pedicle in the midline (Fig. **1**). On the abdominal CT, compared to the previous CT, the stomach was shifted more towards the midline, and the antrum and duodenum were pulled upwards by the pedicled omental flap. The right epigastric vessel, serving as the feeding vessel, was observed passing through the tunnel directly beneath the xiphoid process, accompanying the omentum (Fig. **2**).

### 2.3 Recurrence Case (Case 8)

**Fig. (3):**
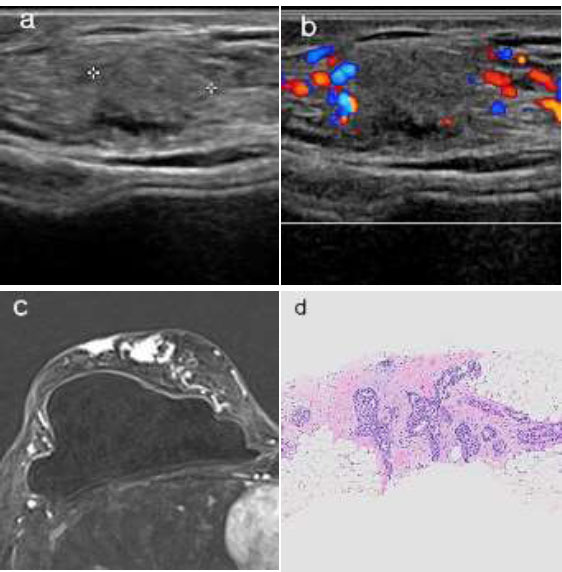
A case of recurrence in the right breast following LHPOF. (**a**, **b**) On ultrasonography, an approximately 1 cm heterogeneous isoechoic mass with indistinct margins is observed, with no vascularity detected relative to the omentum. (**c**) On MRI, the subtraction 2-minute sequence shows multifocal avidly enhancing nodules in the right breast. (**d**) The specimen from the ultrasonography-guided core needle biopsy shows atypically hyperplastic ductal cells in a background of omental adipose tissue [Hematoxylin and Eosin stain, magnification ×40].

The patient was a 35-year-old female who was diagnosed with invasive ductal carcinoma in her right breast. She underwent a skin-sparing mastectomy with LHPOF and implant placement two years ago. The patient underwent four cycles of adjuvant adriamycin and cyclophosphamide chemotherapy postoperatively. After the two-year surveillance follow-up, ill-defined homogeneous, slightly hypoechoic nodules were found on ultrasonography. In addition to this lesion, several other heterogeneous hypoechoic lesions with indistinct margins from the surrounding tissue were observed on ultrasound. An ultrasound-guided 14-G core-needle biopsy revealed invasive ductal carcinoma. Pathology results revealed cancer cells in the background of omental adipose tissue. A subtracted 2-minute sequence of MRI showed scattered heterogeneous enhancing nodules in the right omental flap (Fig. **3**).

## 4 DISCUSSION

The greater omentum, whose blood is provided by the right and left gastroepiploic arteries, is a large apron-like fold of visceral peritoneum that contributes to the peritoneal defense mechanism [6]. The omental flap is a unique flap due to its protective and beneficial roles against infection and its regenerative properties in ischemia. The omental flap can also be easily shaped to fill various defects. Thus, it is widely used in various types of reconstruction procedures, such as fistula closure and head and neck defects [7-9]. However, the current reservations about using the omentum are due to the difficulty in predicting its volume pre-operatively and interpatient variability in size [10, 11]. Moreover, the unique complications involving and affecting abdominal organssuch as an increased incidence of hernias or damage to the abdominal viscera,though rare, may present with their own set of limitations for certain patient populations [12]. Due to the small sample cases in our study, no significant complications occurred.

Previous studies have shown that LHPOF is safe, minimally invasive, and yields good aesthetic outcomes. In a retrospective analysis with a 12-year follow-up, a single-center study of 300 cases [5] and an experienced study of 129 cases reported relatively little donor site deformity, no size reduction after radiation therapy, and oncologic safety [13, 14].

To the best of our knowledge, this is the first study to report imaging findings of patients who underwent LHOF for breast reconstruction. The reconstructed breast exhibits contour almost identical to the contralateral breast on imaging exams. When the contralateral breast is predominantly fatty with minimal glandular tissue, it becomes particularly difficult to discern whether a breast has undergone reconstruction based solely on its imaging characteristics. On abdominal CT, the steeper and more angular contour of the stomach could be related to symptoms of abdominal discomfort, as suggested by previous studies. These changes in the positioning and shape of the stomach may be due to the location and movement of the omental flap, potentially leading to long-term abdominal discomfort for patients. Therefore, long-term follow-up examinations are necessary to monitor these potential complications.

The omentum has many tiny blood vessels and an abundant lymphatic supply. It has been hypothesized that the omentum may promote tumor proliferation and metastasis, as adipocytes provide fatty acids that play a role in tumor growth [15]. Tumor cells convert adipocytes into Cancer-Associated Adipocytes [CAAs], driving a metabolic symbiosis. Cancers stimulate adipocyte lipolysis, releasing Fatty Acids [FAs] that cancer cells absorb *via* receptors like CD36 and FATP1, using them for membrane synthesis, energy, or signaling [16]. Despite this potential for tumor progression, a systematic review of omental flap reconstruction surgeries has shown a very low rate of tumor recurrence, indicating that the clinical risk associated with this procedure is likely minimal. This suggests that while the interaction between cancer cells and adipocytes is significant, it may not pose a substantial threat in the context of omental flap reconstructions [17].

By employing an array of deep learning-based segmentation techniques to quantitatively analyze the omentum and breast volumes before surgery, it becomes possible to more accurately determine the required amount of omental tissue [18, 19]. In doing so, this approach facilitates a more balanced overall breast volume and shape, ultimately resulting in improved aesthetic outcomes.

## CONCLUSION

LHPOF offers a viable, minimally invasive, and cosmetically favorable option for breast reconstruction, with imaging findings closely matching those of the contralateral breast. This method also ensures the effective restoration of tissues. Our study of eight cases in which an LHPOF was used for breast reconstruction post-mastectomy reports the pertinent imaging findings. The reconstructed breasts consistently showed fat replacement of glandular tissue and numerous internal vessels on mammography, ultrasonography, and MRI.

## Figures and Tables

**Table 1:** Characteristics of patients.

S.No.	Age [year]	Side	Type of Breast Surgery	Type of Axillary Surgery	Pathology	Implant	Recurrence	ER	PR	Her2	Ki-67	T Stage	N Stage	Tumor Size	Operation Time
1	35	Rt	NSM	SNB	IDC	YES	YES	8	6	1	20%	T1	N0	6.5cm	7h 30min
2	38	Rt	NSM	ALND	IDC	NO	NO	0	0	0	30%	T2	N1	2.5cm	9h 30min
3	37	Lt	NSM	SNB	IDC	NO	NO	0	0	1	70%	T0	N0	1.4cm	9h 30min
4	38	Rt	NSM	SNB	IDC	YES	NO	8	8	2	10%	T2	N1	3.5cm	9h 15min
5	45	Rt	NSM	SNB	IDC	YES	NO	3	3	3	40%	T1a	N0	3cm	9h
6	41	Lt	NSM	SNB	DCIS	NO	NO	8	8	0	10%	Tis	N0	3.6cm	8h 30min
7	47	Rt	NSM	SNB	DCIS	NO	NO	6	0	1	10%	T1a	N0	0.4cm	8h
8	25	Rt	NSM	SNB	DCIS	NO	NO	8	8	3	10%	T1mi	N1	5.2cm	3h + 3h 40min

**Abbreviations:** **Rt**, right; **Lt,** left; **NSM**, nipple sparing mastectomy; **SNB**, Sentinel lymph node biopsy; **IDC**, invasive ductal carcinoma; **DCIS**, ductal carcinoma *in situ*; **ALND**, axillar lymph node dissection; **ER**, estrogen receptor; **PR**, progesterone receptor; **Her 2**, Human epidermal growth factor receptor 2.

## Data Availability

The data and supportive information are available within the article from the corresponding author [M.B] on reasonable request.

## LIST OF ABBREVIATIONS

LHPOF: Laparoscopically Harvested Pedicled Omental Flap
MRI: Magnetic Resonance Imaging
CT: Computed Tomography
CAA: Cancer-Associated Adipocytes

## STANDARDS OF REPORTING

CARE guidelines were followed.
